# Sebelipase alfa for lysosomal acid lipase deficiency: 5‐year treatment experience from a phase 2 open‐label extension study

**DOI:** 10.1111/liv.14603

**Published:** 2020-08-09

**Authors:** Vĕra Malinová, Manisha Balwani, Reena Sharma, Jean‐Baptiste Arnoux, John Kane, Chester B. Whitley, Sachin Marulkar, Florian Abel

**Affiliations:** ^1^ Department for Metabolic Diseases Children's Clinic General Faculty Hospital and First Faculty of Medicine of Charles University in Prague Prague Czech Republic; ^2^ Department of Genetics and Genomic Sciences and Department of Medicine Icahn School of Medicine at Mount Sinai New York NY USA; ^3^ Department of Endocrinology and Metabolic Medicine Salford Royal Foundation NHS Trust Salford UK; ^4^ Department of Inherited Metabolic Diseases Hôpital Necker‐Enfants Malades Paris France; ^5^ Department of Medicine University of California San Francisco San Francisco CA USA; ^6^ Advanced Therapies Program, and Gene Therapy Center University of Minnesota Minneapolis MN USA; ^7^ Alexion Pharmaceuticals, Inc. Boston MA USA

**Keywords:** enzyme replacement therapy, lipids, liver, lysosomal storage diseases, transaminases

## Abstract

**Background and Aims:**

Lysosomal acid lipase deficiency is characterized by hepatomegaly and dyslipidaemia, which can lead to cirrhosis and premature atherosclerosis. Sebelipase alfa is an approved recombinant human lysosomal acid lipase. In an open‐label extension study of adults with lysosomal acid lipase deficiency (LAL‐CL04), sebelipase alfa treatment for 1 year reduced serum transaminase levels and liver fat content and improved serum lipid levels.

**Methods:**

Final data from LAL‐CL04 are reported herein for patients who received sebelipase alfa infusions (1.0 or 3.0 mg/kg every other week) for up to 5 years.

**Results:**

Of 8 patients enrolled, 7 received sebelipase alfa for 224‐260 weeks; 1 was lost to follow‐up. Median baseline levels of alanine aminotransferase and aspartate aminotransferase (81.5 and 50.0 U/L, respectively) were decreased through the end‐of‐study visit (54.0 and 34.0 U/L). Median low‐density lipoprotein cholesterol decreased from 113 to 78 mg/dL, total cholesterol decreased from 171 to 132 mg/dL, and high‐density lipoprotein cholesterol increased from 37 to 42 mg/dL. Most treatment‐emergent adverse events were nonserious (99%), mild/moderate (98%) and unrelated to sebelipase alfa (87%); no patient discontinued as a result of treatment‐emergent adverse events. One patient had 2 serious treatment‐emergent adverse events (cholecystitis and cholelithiasis; assessed as unlikely related to sebelipase alfa). Two patients had 20 nonserious infusion‐associated reactions in weeks 6‐38; all were manageable. One patient tested positive for antidrug antibodies (single occurrence).

**Conclusions:**

Sebelipase alfa was well tolerated and improved serum transaminase and lipid levels for up to 5 years in adults with lysosomal acid lipase deficiency.

**Trial registration number:** ClinicalTrials.gov record NCT01488097.

**Abbreviations**
ADAs: antidrug antibodies
AE: adverse event
ALT: alanine aminotransferase
APRI: AST‐to‐platelet ratio index
AST: aspartate aminotransferase
ECG: electrocardiogram
FIB4: fibrosis‐4 index
GGT: γ‐glutamyltransferase
HDL‐C: high‐density lipoprotein cholesterol
hs‐CRP: high‐sensitivity C‐reactive protein
IAR: infusion‐associated reaction
LAL: lysosomal acid lipase
LDL‐C: low‐density lipoprotein cholesterol
MEGE‐MRI: multi‐echo gradient‐echo magnetic resonance imaging
MN: multiples of normal
MRS: ^1^H‐magnetic resonance spectroscopy
qow: every other week
qw: once weekly
SA: sebelipase alfa
TEAE: treatment‐emergent adverse event
ULN: upper limit of normal

**Key points**

- Lysosomal acid lipase (LAL) deficiency is a rare, inherited lysosomal storage disorder that can affect people of all ages and has a highly variable clinical presentation.
- Sebelipase alfa, a recombinant human LAL approved for the treatment of LAL deficiency, improves markers of liver cell injury and LAL deficiency–related lipid abnormalities; in infants, who have the most rapidly progressive form of the disease, sebelipase alfa prolongs survival.
- This study evaluated the efficacy and safety of sebelipase alfa in adults with liver dysfunction as a result of LAL deficiency who were treated for up to 5 years.

## 1 INTRODUCTION

In lysosomal acid lipase (LAL) deficiency, functional mutations in the *LIPA* gene lead to a complete or nearly complete absence of LAL activity. The consequence is lysosomal accumulation of triglycerides and cholesteryl esters in a variety of cell types and tissues (eg hepatocytes, macrophages, endothelial cells).^1^, ^2^, ^3^, ^4^ Diagnosis is confirmed by measuring LAL activity in dried blood spots, leukocytes or fibroblasts.^1^, ^3^, ^5^, ^6^, ^7^

The clinical sequelae of LAL deficiency in children and adults vary, and may include liver disease with hepatomegaly, elevated serum transaminases, microvesicular steatosis and fibrosis or cirrhosis.^4^, ^8^ Altered lipid metabolism leads to elevated low‐density lipoprotein cholesterol (LDL‐C) levels and low high‐density lipoprotein cholesterol (HDL‐C) levels, with accelerated vascular disease of variable degree.^1^, ^3^, ^4^, ^9^, ^10^

Dietary modification and lipid‐modifying medications are not effective in treating and preventing the progression of disease and neither addresses the underlying pathology. Liver transplantation is not well studied in this population and is associated with numerous complications, including the need for further transplantation.^3^, ^4^, ^8^, ^11^ Sebelipase alfa (SA) is a recombinant human enzyme replacement therapy indicated for the treatment of patients with LAL deficiency.^12^, ^13^ In the first human study of SA (LAL‐CL01), a phase 1/2, open‐label, multicenter dose‐escalation study, 9 adults with LAL deficiency received 4 once‐weekly (qw) infusions of SA (0.35, 1.0 or 3.0 mg/kg).^14^ Patients who completed LAL‐CL01 were eligible to enrol in an open‐label, multicenter, phase 2 extension study (LAL‐CL04), in which patients received 4 qw infusions of SA (0.35, 1.0 or 3.0 mg/kg), followed by every‐other‐week (qow) SA infusions of 1.0 mg/kg (for patients previously treated with 0.35 or 1.0 mg/kg qw) or 3.0 mg/kg (for patients previously treated with 3.0 mg/kg qw). By 1 year, patients had sustained reductions in serum transaminase levels and liver volume and fat content, and improvements in serum lipid levels.^15^

The objective of this analysis was to assess the efficacy and safety of SA for the treatment of adults with liver dysfunction as a result of LAL deficiency who received SA for up to 5 years in study LAL‐CL04.

## 2 PATIENTS AND METHODS

### 2.1 Patients

Eligibility criteria for LAL‐CL01 were described previously.^14^ Patients eligible for LAL‐CL04 were men and women ≥18 years of age who received all 4 scheduled doses of SA in study LAL‐CL01 with no life‐threatening or unmanageable toxicity; patients were willing and able to comply with study procedures and provided written informed consent. Females were required to have a negative serum pregnancy test result at screening, were not breastfeeding and agreed to use an approved contraceptive method from the time of informed consent until 30 days after the last SA dose. Patients were excluded for clinically significant concurrent disease, serious intercurrent illness, use of concomitant medications or extenuating circumstances that, in the opinion of the investigator, would interfere with participation or interpretation of results. Patients were also excluded for clinically significant abnormal values on laboratory screening tests other than liver function or lipid panel tests.

### 2.2 Study design

This was a phase 2, open‐label extension study carried out at 11 sites in the United States, United Kingdom, France, the Czech Republic and Canada (ClinicalTrials.gov record NCT01488097) between December 2011 and June 2017. Patients initiated treatment in LAL‐CL04 at least 4 weeks after the last SA dose in LAL‐CL01. The starting dose in LAL‐CL04 was the same qw dose that the patient received in LAL‐CL01 (0.35, 1.0, or 3.0 mg/kg [n = 3 in each group in LAL‐CL01]), continued for 4 weeks.^14^ At week 6, all patients transitioned to qow doses (1.0 mg/kg if previously receiving 0.35 or 1.0 mg/kg qw; 3.0 mg/kg if previously receiving 3.0 mg/kg qw) infused over approximately 2 hours. Infusions were administered at the study site or an approved local medical centre. Patients in the United States who were on a stable dose of SA for at least 12 months with no infusion‐associated reactions (IARs) that required medical intervention or management and no treatment‐related serious adverse events during the prior 6 months were eligible to receive infusions at home. Patients approved for home infusion were to return to the study site for scheduled assessments. Treatment duration in LAL‐CL04 was up to 5 years. The last follow‐up visit was approximately 30 days after the last SA dose.

Dose increases from 1.0 mg/kg to 3.0 mg/kg qow and from 3.0 mg/kg qow to 3.0 mg/kg qw were allowed for inadequate clinical response (defined as clinically important LAL deficiency manifestations on clinical examination, laboratory assessment, liver biopsy or imaging that did not improve from baseline, improved and plateaued but did not normalize, or failed to normalize within 12 months of treatment initiation). Patients who did not tolerate their SA dose could receive a dose reduction to the next lowest level (from 3.0 to 1.0 mg/kg qow; from 1.0 to 0.35 mg/kg qow) at the investigator's discretion. If a patient did not tolerate 0.35 mg/kg qow, despite measures to manage IARs, the patient was discontinued from the study.

The study was conducted according to the International Council for Harmonisation Good Clinical Practice guidelines. The study protocol conforms to the ethical guidelines of the 1975 Declaration of Helsinki and received a priori approval by each institution's human research committee in agreement with local legal requirements. The authors followed the STROBE cohort guidelines in reporting this study.^16^

### 2.3 Efficacy assessments

Absolute changes from baseline in serum alanine aminotransferase (ALT) and aspartate aminotransferase (AST) levels were evaluated. The upper limit of normal (ULN) was 67 U/L for ALT and 50 U/L for AST. Other disease‐relevant assessments included absolute changes from baseline in lipids (total cholesterol, triglycerides, LDL‐C and HDL‐C), γ‐glutamyltransferase (GGT), bilirubin, albumin, haemoglobin, platelets, ferritin and high‐sensitivity C‐reactive protein (hs‐CRP). The AST‐to‐platelet ratio index (APRI), calculated as [(AST level/AST ULN)/platelet count] × 100, and the fibrosis‐4 (FIB4) index, calculated as (age at start of infusion × AST level)/(platelet count × √ALT level), were evaluated as surrogate biomarkers of liver fibrosis/cirrhosis.

Multi‐echo gradient‐echo magnetic resonance imaging (MEGE‐MRI) and/or ^1^H‐magnetic resonance spectroscopy (MRS) were used to measure changes from baseline in liver and spleen volume (measured in multiples of normal [MN]; normal = 2.5% of body weight [for liver measurements] and 0.2% of body weight [for spleen measurements]) and liver and spleen fat content. To ensure consistency, abdominal MRI/MRS scans were read centrally at Biomedical Systems, by readers blinded to the time of image acquisition.

Optional liver biopsies were obtained where possible for hepatic histology evaluation between weeks 52 and 104. If a patient had a liver biopsy prior to screening, available results were recorded in the electronic case report form. Pathology reports for biopsies performed prior to treatment were also provided, if available.

### 2.4 Safety assessments

The incidence of treatment‐emergent adverse events (TEAEs), serious adverse events and IARs was determined. Adverse event (AE) severity was graded on a 5‐point scale (mild, moderate, severe, life‐threatening and death) according to National Cancer Institute Common Terminology Criteria for Adverse Events, version 4.03. All AEs were coded using the Medical Dictionary for Regulatory Activities, version 20.0. IARs were defined as any AE that occurred during the 2‐hour infusion or within 4 hours after the infusion and was assessed as at least possibly related to the study drug.

Testing for antidrug antibodies (ADAs) was routinely performed for all patients, and any patient who tested positive was subsequently tested for the presence of neutralizing antibodies that inhibit SA enzyme activity and/or cellular uptake. Analysis of ADAs and neutralizing antibodies was performed by a central laboratory (Covance).

Vital signs were measured; blood and urine samples were collected for clinical laboratory evaluations; a complete physical examination was conducted and 12‐lead electrocardiograms (ECGs) were obtained at various times throughout the study.

### 2.5 Statistical analyses

Descriptive analyses were performed; data were summarized as median values. Baseline values were the last measurement before the first infusion of SA in study LAL‐CL04. Median change from baseline values is reported for patients who had both baseline and end‐of‐study data. The full analysis set was used for efficacy analyses and consisted of all patients who received at least 1 complete infusion of SA and had at least 1 post‐treatment measurement. The safety analysis set consisted of all patients who received any amount of SA. No formal inferential statistical testing was planned or performed. All analyses were performed with the SAS statistical software system (Life Science Analytics Framework). Statistical analyses were conducted by Alexion Pharmaceuticals, Inc All authors had access to all of the data and vouch for the integrity of the data analyses.

## 3 RESULTS

### 3.1 Patient disposition

Eight of 9 patients who completed study LAL‐CL01 were enrolled in the LAL‐CL04 extension study^15^; 3 patients received a starting dose of SA of 0.35 mg/kg qw, 2 received 1.0 mg/kg qw and 3 received 3.0 mg/kg qw. After 4 infusions, all patients transitioned to a qow dosing regimen; 5 patients received 1.0 mg/kg qow and 3 patients received 3.0 mg/kg qow. Of these 8 patients, 7 received open‐label SA treatment for 224‐260 weeks and completed the study, after which they transitioned to commercial therapy; 1 patient was lost to follow‐up after week 150 (the patient dropped out pending attempts to conceive a child).^15^ The first study participant entered the extension study on December 12, 2011 and the last participant completed the study on June 21, 2017. All 8 patients initiated treatment between approximately 9 and 28 weeks after their last dose in study LAL‐CL01. All were white, 6 were men and 7 were nonobese (body mass index <30.0 kg/m^2^). As previously reported,^15^ 1 patient who completed LAL‐CL01 did not decide to participate in LAL‐CL04 until 10 months after completing therapy with sebelipase alfa in LAL‐CL01. During this 10‐month off‐treatment period, the patient experienced worsening liver disease with evidence of hepatic decompensation and required urgent liver transplantation. These complications made the patient ineligible for LAL‐CL04. Baseline demographic and disease characteristics of the 8 patients who were enrolled in LAL‐CL04 are summarized in Table 1.

**TABLE 1:** Baseline demographic and disease characteristics

Variable	Total (N = 8)	ULN or LLN
Age at LAL‐D diagnosis, y^a^		
Mean (SD)	15.6 (15.7)	
Median	9.1	
Range	4.1‐42.4	
Age at start of SA treatment, y^b^		
Mean (SD)	30.3 (10.7)	
Median	29	
Range	19‐45	
Gender, n (%)		
Male	6 (75)	
Female	2 (25)	
White race, n (%)	8 (100)	
Body mass index, kg/m^2^		
Mean (SD)	25.7 (3.4)	
Median	26.1	
Range	19.9‐31.3	
ALT		
Median (range), U/L	81.5 (36‐109)	ULN = 67 U/L
>1.5 × ULN, n (%)	1 (13)	
AST		
Median (range), U/L	50.0 (29‐64)	ULN = 50 U/L
>1.5 × ULN, n (%)	0 (0)	
GGT		
Median (range), U/L	29.5 (14‐148)	ULN = 73 U/L
TC		
Median (range), mg/dL	170.7 (103‐311)	ULN = 232 mg/dL
TG		
Median (range), mg/dL	104.1 (54‐336)	ULN = 199 mg/dL
LDL‐C		
Median (range), mg/dL	113.1 (66‐297)	ULN = 162 mg/dL
HDL‐C		
Median (range), mg/dL	36.9 (19‐51)	LLN = 35 mg/dL
Liver volume, MN, median (range)^c^	1.00 (0.87‐1.24)	
Liver fat content, %, median (range)^d^	10.41 (4.00‐12.29)	

Abbreviations: ALT, alanine aminotransferase; AST, aspartate aminotransferase; GGT, γ‐glutamyltransferase; HDL‐C, high‐density lipoprotein cholesterol; LAL‐D, lysosomal acid lipase deficiency; LDL‐C, low‐density lipoprotein cholesterol;LLN, lower limit of normal; MN, multiples of normal; SA, sebelipase alfa; TC, total cholesterol; TG, triglycerides; ULN, upper limit of normal.

^a^ As reported in study LAL‐CL01.

^b^ Start of study LAL‐CL01.

^c^ Normal = 2.5% of body weight.

^d^ n = 5.

### 3.2 Efficacy

#### 3.2.1 Serum transaminases

Improvements in serum ALT and AST levels were maintained through the end‐of‐study visit. Table 2 shows absolute values at baseline and end of study and changes from baseline to end of study.

**TABLE 2:** Change from baseline to end of study for key efficacy assessments

Parameter	Baseline	EOS	Median change from baseline to EOS^a^	ULN or LLN
ALT	n = 8	n = 7	n = 7	
Median (range), U/L	81.5 (36‐109)	54.0 (24‐80)	−43.0 (−63 to 18)	ULN = 67 U/L
AST	n = 8	n = 7	n = 7	
Median (range), U/L	50.0 (29‐64)	34.0 (23‐47)	−16.0 (−30 to 9)	ULN = 50 U/L
GGT	n = 8	n = 7	n = 7	
Median (range), U/L	29.5 (14‐148)	28.0 (7‐134)	−4.0 (−14 to 10)	ULN = 73 U/L
TC	n = 8	n = 7	n = 7	
Median (range), mg/dL	170.7 (103‐311)	132.3 (99‐250)	−20.1 (−135 to 68)	ULN = 232 mg/dL
TG	n = 8	n = 7	n = 7	
Median (range), mg/dL	104.1 (54‐336)	112.5 (52‐287)	−1.8 (−161 to 166)	ULN = 199 mg/dL
LDL‐C	n = 8	n = 7	n = 7	
Median (range), mg/dL	113.1 (66‐297)	78.1 (54‐187)	−20.1 (−123 to 9)	ULN = 162 mg/dL
HDL‐C	n = 8	n = 7	n = 7	
Median (range), mg/dL	36.9 (19‐51)	42.2 (21‐70)	8.1 (−5 to 19)	LLN = 35 mg/dL
Liver volume	n = 8	n = 5	n = 5	
Median (range), MN^b^	1.00 (0.87‐1.24)	0.82 (0.71‐1.13)^c^	−0.17 (−0.26 to −0.11)	
Liver fat content	n = 5	n = 4	n = 3	
Median (range), %	10.41 (4.00‐12.29)	3.47 (1.30‐4.12)^c^	−3.91 (−7.48 to −0.63)	

Abbreviations: ALT, alanine aminotransferase; AST, aspartate aminotransferase; EOS, end of study; GGT, γ‐glutamyltransferase; HDL‐C, high‐density lipoprotein cholesterol; LDL‐C, low‐density lipoprotein cholesterol; LLN, lower limit of normal; MN, multiples of normal; TC, total cholesterol; TG, triglycerides; ULN, upper limit of normal.

^a^ Median change from baseline values is reported for only those patients who had both baseline and end‐of‐study data.

^b^ Normal = 2.5% of body weight.

^c^ Week 208 (last time point with n >2).

**FIGURE 1:**
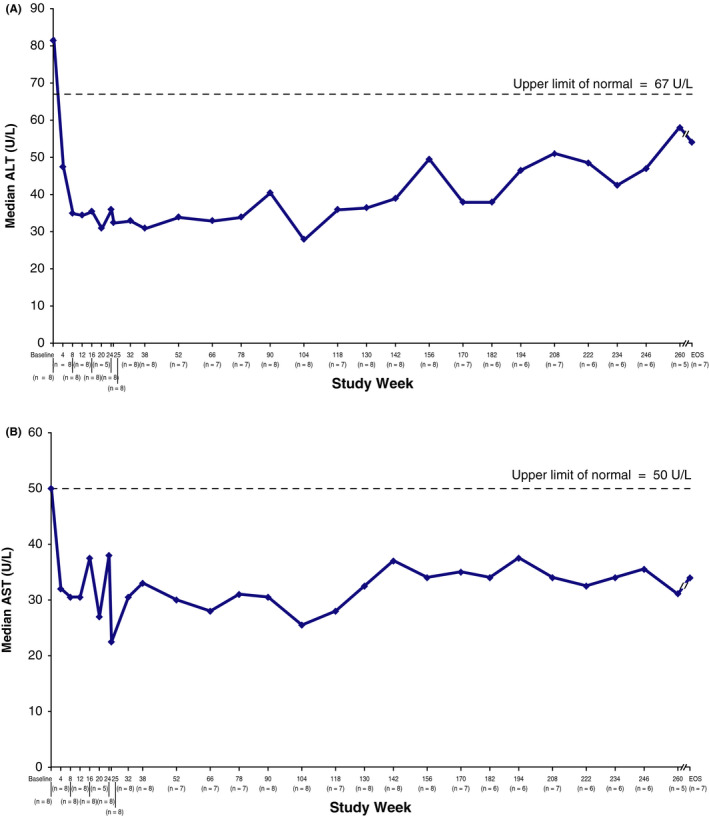
Changes in median ALT (A) and AST (B) over time. Data were collected every 4 weeks through week 24, at weeks 25, 32, 38, and every 12‐14 weeks thereafter (weeks 52, 66, 78, 90, 104, 118, 130, 142, 156, 170, 182, 194, 208, 222, 234, 246, 260), and at end of study (EOS). Patients were allowed to schedule week 25 visit at week 25, 27 or 29. ALT, alanine aminotransferase; AST, aspartate aminotransferase

Figure 1 shows serum ALT and AST profiles over time. Serum transaminase levels normalized during treatment for all patients who had abnormal levels at baseline. Of the 5 patients with baseline ALT exceeding the ULN (67 U/L), 4 achieved normal levels by week 4 and all 5 had normal ALT levels by week 8. All 4 patients with baseline AST exceeding the ULN (50 U/L) achieved normal AST levels by week 4. Thereafter, ALT and AST levels generally remained within the normal range, although occasional abnormal values were reported in individual patients.

#### 3.2.2 Serum lipids

**FIGURE 2:**
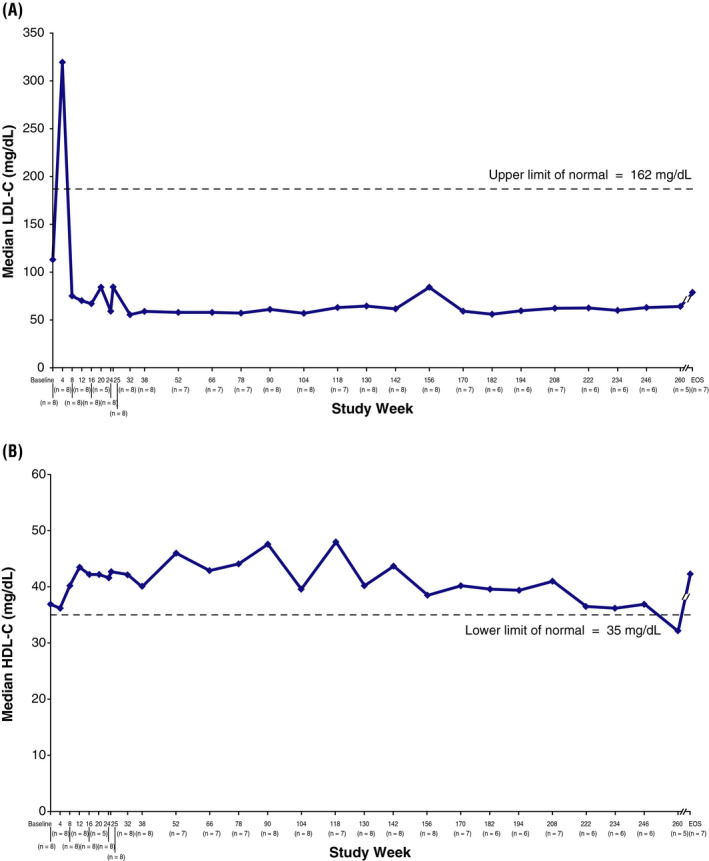
Changes in median LDL‐C (A) and HDL‐C (B) over time. Data were collected every 4 weeks through week 24, at weeks 25, 32, 38, and every 12‐14 weeks thereafter (weeks 52, 66, 78, 90, 104, 118, 130, 142, 156, 170, 182, 194, 208, 222, 234, 246 and 260) and at end of study (EOS). Patients were allowed to schedule week 25 visit at week 25, 27 or 29. HDL‐C, high‐density lipoprotein cholesterol; LDL‐C, low‐density lipoprotein cholesterol

Improvements in serum lipids were also sustained with SA treatment. Absolute values at baseline and end of study and changes from baseline to end of study are shown in Table 2. Serum LDL‐C and HDL‐C profiles over time are shown in Figure 2. Five patients (63%) were receiving concomitant lipid‐modifying medications at baseline (statins). In addition, ezetimibe, cholestyramine, nicotinic acid and omega‐3‐acid ethyl esters were each administered to 1 (13%) patient. During the study, 1 of the 3 patients not receiving lipid‐modifying medications at LAL‐CL04 baseline‐initiated simvastatin at approximately week 40. This patient had been receiving lipid‐modifying medication during LAL‐CL01, but discontinued the medication between LAL‐CL01 and LAL‐CL04. The patient continued simvastatin treatment for the duration of the study, with a few brief interruptions. Another patient receiving simvastatin had a dose reduction at week 91 and discontinued simvastatin treatment at week 182.

#### 3.2.3 Liver volume and fat content

Liver volume and fat content decreased from baseline to week 208 (the last assessment at which data were available for >2 patients). Absolute values at baseline and week 208 and changes from baseline to week 208 are shown in Table 2. A reduction in median liver volume from the baseline value of 1.00 MN (n = 8) was apparent by week 10/12 (median change from baseline, −0.10 MN; n = 8), and by week 104 liver volume had decreased in all 7 patients with available data (median change from baseline, −0.12 MN). This improvement was maintained during long‐term SA treatment; at week 208, the median liver volume was 0.82 MN (median change from baseline, −0.17 MN; n = 5).

**FIGURE 3:**
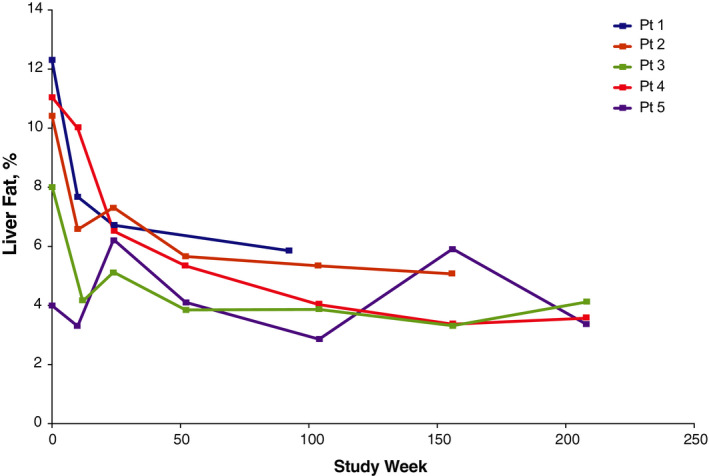
Change in liver fat content over time. Assessed by multi‐echo gradient‐echo magnetic resonance imaging of right lobe. Data were collected at baseline, weeks 10 or 12, 24, 52, 104, 156, 208, 260, and end of study (30 days after last dose of sebelipase alfa with a +7‐day window); week 208 = last time point with n >2. The final assessment for patient 1 was an unscheduled visit at week 92. Pt, patient

A reduction in median liver fat content from the baseline value of 10.41% (n = 5) was apparent by week 10/12 (median change from baseline, −3.84%; n = 5); further reductions were observed through week 104 (median change from baseline, −4.62%; n = 4), and improvement was maintained for the duration of long‐term treatment. At week 208, median liver fat content was 3.47% (n = 4; median change from baseline, −3.91% [n = 3]). Figure 3 illustrates individual patient data for changes in liver fat content over time for the 5 patients with both pre‐ and post‐treatment results available.

#### 3.2.4 Spleen volume and fat content

At baseline, the median spleen volume was 2.09 MN (range, 1.62‐3.15 MN; n = 8), and at week 208 (the last assessment at which data were available for >2 patients), it was 2.11 MN (range, 1.20‐3.17 MN; n = 5). The median change in spleen volume from baseline to week 208 was −0.11 MN (range, −0.5 to 0.18 MN; n = 5 patients with paired data). No clear trend towards an improvement in spleen volume was observed during SA treatment. Both increases and decreases were observed for individual patients at each time point from week 10/12 through week 208. Median baseline spleen fat content was 1.34% in the 5 patients with available MEGE‐MRI data (range, 0.77%‐2.16%); median spleen fat content at week 208 was 1.20% (range, 0.28%‐1.29%; n = 3). All 5 patients had apparent reductions in spleen fat content over time. The median change in spleen fat content from baseline to week 208 was −0.68% (range, −0.68% to 0.43%; n = 3 patients with paired data).

#### 3.2.5 Other parameters

Regarding other liver biochemical parameters, although the median serum GGT level decreased from baseline (29.5 U/L) at week 4 (median change, −5.5 U/L) and through the end of the study (median change, −4.0 U/L), this was owing largely to 1 patient with a markedly elevated baseline level that decreased rapidly on treatment. GGT levels of the other 7 patients were normal at baseline and through end of study. Median total bilirubin was 0.94 mg/dL at baseline; a decrease at week 4 (median change, −0.31 mg/dL) was sustained through end of study (median change, −0.19 mg/dL). The median albumin level, 42.3 g/L at baseline, increased slightly during treatment to 47.0 g/L at end of study (median change from baseline, 5.0 g/L).

Most patients had normal values for haematology and coagulation parameters at baseline and throughout the study. No trends in individual shifts between normal and abnormal values were apparent from baseline to each time point for any parameter. The median haemoglobin level was 154.5 g/L at baseline and 155.5 g/L at end of study. Median platelet counts at baseline and end of study were 208 × 10^9^/L and 191 × 10^9^/L respectively. The median serum ferritin level was 158 µg/L at baseline and 163 µg/L at end of study, and median hs‐CRP was 1.30 mg/L at baseline and 1.80 mg/L at end of study.

The median APRI decreased from 0.445 at baseline (N = 8) to 0.348 at end of study (n = 6), with a median change from baseline to end of study of −0.186 (n = 6 patients with paired data). The median FIB4 changed from 0.654 at baseline (N = 8) to 0.663 at end of study (n = 6), with a median change from baseline at end of study of −0.234 (n = 6 patients with paired data).

#### 3.2.6 Liver biopsies

**FIGURE 4:**
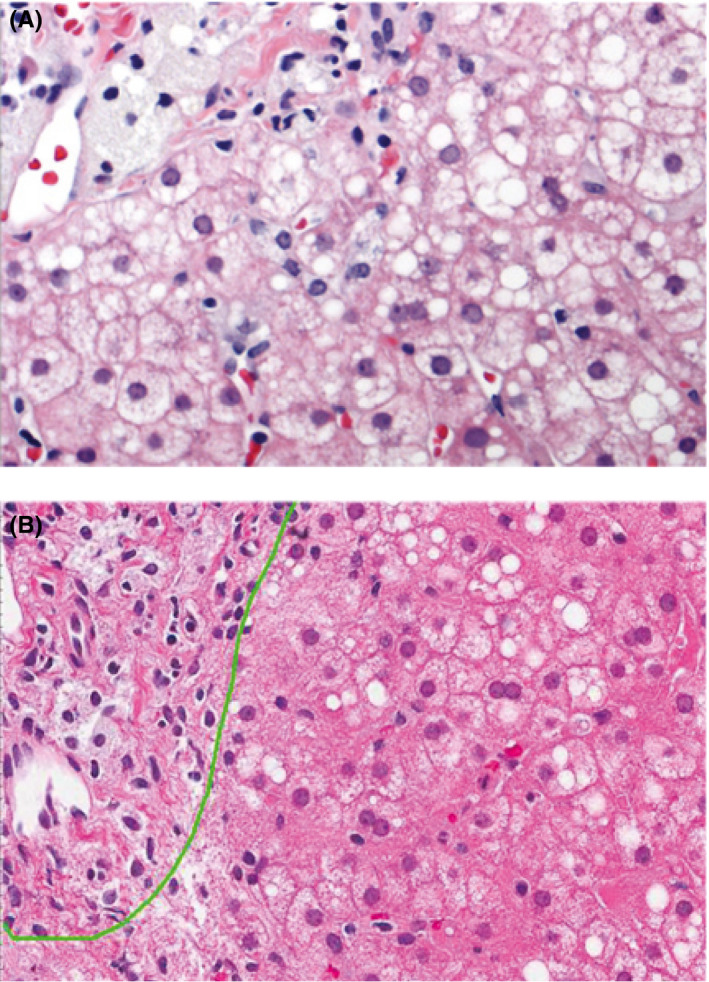
Liver biopsy findings in 1 patient with pre‐ and post‐treatment assessments. Pretreatment biopsy (A) shows microvesicular steatosis with fibrosis. Post‐treatment biopsy approximately 2 years after sebelipase alfa treatment initiation (B) shows mild microvesicular steatosis with no significant fibrosis

Two patients underwent pre‐ and post‐treatment liver biopsies. The first patient was a man diagnosed with LAL deficiency at 39 years of age who received his first SA dose in LAL‐CL01 on May 10, 2011 (at 41 years of age). A biopsy in December 2007 showed microvesicular steatosis with fibrosis (Figure 4A); a biopsy in 2013, approximately 2 years after SA treatment initiation, showed mild microvesicular steatosis with no significant fibrosis (Figure 4B).

**FIGURE 5:**
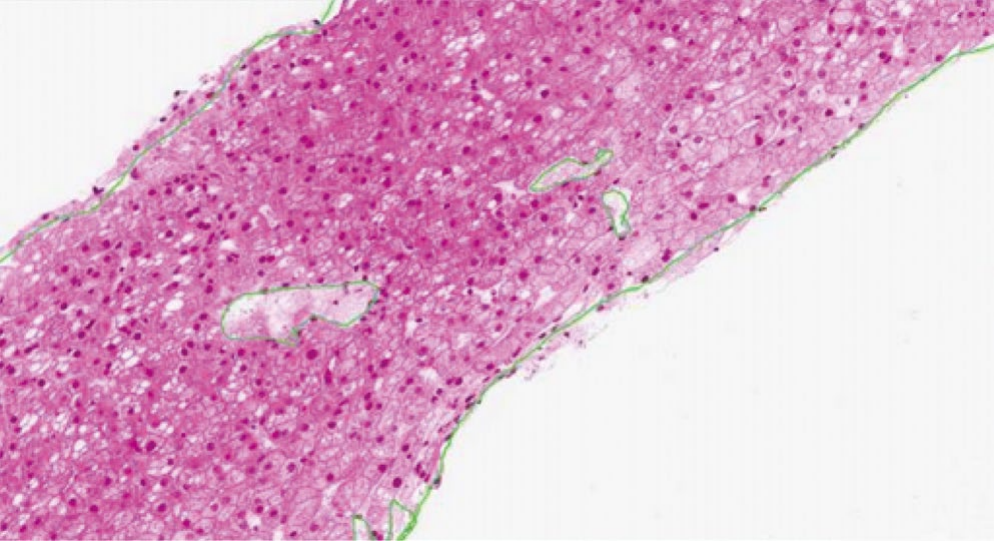
Liver biopsy findings in 1 patient with post‐treatment assessment approximately 1.5 years after sebelipase alfa treatment initiation showing steatosis with mild microvesicular predominance (20%‐30%) and periportal fibrosis with some septa (F2 Metavir score)

The second patient was a young man diagnosed with LAL deficiency at 12 years of age who had his first SA dose in LAL‐CL01 on October 6, 2011 (at age 20). A biopsy taken in January 2009 showed micro‐ and macrovesicular steatosis (predominantly microvesicular) affecting 80% of hepatocytes, and secondary macrophage and hepatocyte lipid overloading with extensive fibrosis, with numerous fibrous bridges and incipient nodulations. A biopsy in July 2013, approximately 1.5 years after SA treatment initiation, showed steatosis with mild microvesicular predominance (20%‐30%) associated with foamy macrophages and periportal fibrosis with some septa (F2 Metavir score) (Figure 5). There was almost no other histological inflammation of the sample examined. The patient's prebiopsy elastometry (6.3 ± 1.4 kPa) was considered normal.

### 3.3 Safety

#### 3.3.1 Infusions and concomitant medications

During the extension study, 920 infusions were administered to 8 patients; 5 (63%) patients received all infusions without dose interruption or modification. Of the remaining 3 (38%), 1 had a single modified infusion owing to human error, 1 had a single modified infusion as a result of an accidental rate change plus 1 missed infusion caused by a TEAE of cholecystitis unrelated to treatment and 1 had numerous dose interruptions as a result of mild‐to‐severe, nonserious IARs, but resumed treatment using a desensitization protocol that involved stepwise increases in the infusion rate.

Seven (88%) patients were receiving 1 or more medications at baseline, most of which were used for the management of LAL deficiency symptoms and complications or comorbidities, and were continued during the study. All 8 patients received 1 or more concomitant medications during the study, most commonly analgesics (7 [88%]), antibacterial agents (6 [75%]), lipid‐modifying medications (6 [75%]) and vitamins (5 [63%]).

#### 3.3.2 Adverse events

All 8 patients experienced TEAEs (Table 3). Of the 271 TEAEs reported, most were nonserious (99%), mild or moderate in severity (98%) and assessed by the investigator as unrelated or unlikely to be related to SA (87%). The most frequently reported TEAEs (≥50% of patients) were abdominal pain, nausea, viral upper respiratory tract infection, back pain and diarrhoea. Related or possibly related TEAEs were reported in 5 (63%) patients. One patient had 2 serious AEs (cholecystitis and cholelithiasis) assessed as unlikely to be related to SA. No withdrawals owing to TEAEs occurred, and no deaths were reported.

**TABLE 3:** Patients experiencing an adverse event (N = 8)

Event	n (%)
Any TEAE	8 (100)
Most common TEAEs (>2 patients)	
Abdominal pain	5 (63)
Nausea	5 (63)
Viral upper respiratory tract infection	5 (63)
Back pain	4 (50)
Diarrhoea	4 (50)
Ear pain	3 (38)
Abdominal pain upper	3 (38)
Fatigue	3 (38)
Musculoskeletal pain	3 (38)
Myalgia	3 (38)
Oropharyngeal pain	3 (38)
Skin mass	3 (38)
TEAEs related or possibly related to SA^a^	5 (63)
IARs	2 (25)
Any treatment‐emergent SAEs^b^	1 (13)
Any related or possibly related treatment‐emergent SAEs	0 (0)
Dose modification owing to TEAE	2 (25)
Discontinued study owing to TEAE	0 (0)
Deaths	0 (0)

Abbreviations: IAR, infusion‐associated reaction; SA, sebelipase alfa; SAE, serious adverse event; TEAE, treatment‐emergent adverse event.

^a^ Abdominal pain, fatigue, lethargy, keratitis, sunburn, dry eyes, worsening acne and hypertension (n = 1); soft faeces, hyperaemia, chills, laryngeal oedema, abdominal pain, nausea, dizziness and IAR (n = 1); diarrhoea, abdominal pain, gastro‐oesophageal reflux disease, paresthesia and IAR (n = 1); intrahepatic biliary stones and cholelithiasis (n = 1); abdominal pain (n = 1).

^b^ Cholecystitis and cholelithiasis; assessed as unlikely to be related to sebelipase alfa by the investigator.

Two patients who were siblings had 20 nonserious IARs; all but 2 IARs were mild to moderate in severity, and all were successfully managed by dose interruption or pretreatment with an antihistamine, an antipyretic and subcutaneous epinephrine (for potential laryngeal oedema). Both patients initially experienced IARs at week 6, following their first infusion of SA 1.0 mg/kg, and their last IAR was reported at week 36 or week 38. One of them had SA treatment withheld from week 40 to week 88 to allow further investigation following a moderate IAR. The patient successfully restarted SA at week 90 under a desensitization protocol, initially at a reduced dose (0.35 mg/kg qow) and with pretreatment with diphenhydramine 50 mg and ibuprofen 400 mg prior to SA infusions from weeks 90 to 100, and diphenhydramine 25 mg and acetaminophen 650 mg prior to SA infusions from weeks 102 to 116. Beginning with the week 98 infusion, the SA dose was returned to the previous dose of 1.0 mg/kg qow. By week 118, the patient was receiving infusions at the planned dose (1.0 mg/kg qow) and infusion rate, and without pretreatment. The patient received SA infusions through week 150 and was subsequently lost to follow‐up pending attempts to conceive a child. The second patient experienced mild‐to‐moderate IARs and continued in the study without dose interruption or pretreatment through study completion.

#### 3.3.3 Immunogenicity

One patient tested positive for ADAs at a single time point (week 4), with a low titre of 80. This result was considered unlikely to represent a true seroconversion because the patient's subsequent ADA test results were negative. This patient also tested negative for neutralizing antibodies in both the enzyme activity and cell uptake assays. No IARs or other treatment‐related TEAEs were reported for this patient; thus, no conclusions can be made regarding the impact of ADAs on the safety profile of SA.

#### 3.3.4 Changes in vital signs, physical examination findings, clinical laboratory tests, and ECG parameters

No clinically meaningful trends were observed in systolic or diastolic blood pressure, heart rate, respiratory rate or body temperature, either in association with SA infusions or over the course of the study, based on maximum increases and decreases from pre‐infusion values at each visit. General physical examination findings at screening were consistent with those expected in this patient population. Serum chemistry parameters evaluated as safety outcomes were within the normal range in most patients at baseline and remained normal throughout the study. No trends in individual shifts between normal and abnormal values were apparent from baseline to each time point. One patient with a high‐normal baseline serum creatinine level (1.18 mg/dL; ULN, 1.20 mg/dL) had a TEAE of increased serum creatinine with onset at approximately week 4 (1.37 mg/dL); this value continued to be elevated intermittently, peaking at week 104 (1.53 mg/dL). This TEAE was considered resolved at week 158, without intervention, and was assessed as moderate and unlikely related to treatment. One patient with markedly elevated hs‐CRP at baseline (11.0 mg/L; ULN, 3.0 mg/L) had a TEAE of increased hs‐CRP (3.7 mg/L) with onset at week 222; hs‐CRP levels were below baseline at all assessments during treatment, although they fluctuated broadly over the 5‐year treatment period (range, 0.3‐10.1 mg/L) and were frequently abnormal. This TEAE was considered resolved at week 234 (hs‐CRP, 2.0 mg/L), and was assessed as mild and unlikely related to treatment. No clinically meaningful trends were apparent over time in urinalysis or ECG parameters (PR, QRS, QT and QTc) based on examination of changes from baseline to each time point.

## 4 DISCUSSION

The first adult human study of SA demonstrated that SA was well tolerated and rapidly reduced serum transaminases and improved lipid profiles in adults with LAL deficiency.^14^ Patients with serum transaminase levels above the ULN at baseline experienced a normalization of serum transaminase levels that was generally sustained for the duration of the study. Occasional transient increases in ALT and AST levels were noted in individual patients. Improvements in lipid profiles and reductions in liver volume and fat content were also sustained. Given the low baseline spleen fat content of these patients, it was not possible to draw firm conclusions about the effect of SA therapy on lipid substrate accumulation in the spleen. No patient demonstrated clinical progression of LAL deficiency during treatment with SA. In addition, treatment with SA for up to 5 years was well tolerated; 98% of TEAEs were mild to moderate in severity and no patient discontinued treatment because of TEAEs. SA treatment was also associated with a low frequency of generally mild‐to‐moderate IARs. Although 1 patient tested positive for ADAs at a single time point, an association between ADAs and clinical efficacy or safety could not be made. These findings are consistent with the SA mechanism of action, in that it effectively replaces the missing LAL enzyme, addressing the underlying pathophysiology of LAL deficiency to achieve favourable effects on the broad range of abnormalities associated with this disease.

The interval between completion of study LAL‐CL01 and enrolment for the 8 patients in this study ranged from 9 to 28 weeks, and during that time off treatment, increases in serum transaminases and LDL‐C and decreases in HDL‐C were observed relative to levels at the end‐of‐study LAL‐CL01,^15^ highlighting the benefit of continuous SA treatment. As previously reported, the 1 patient who did not participate in LAL‐CL04 experienced rapidly progressive liver dysfunction requiring urgent liver transplantation during the 10 months after completing study LAL‐CL01,^15^ further highlighting the potential for rapid deterioration in the absence of treatment, even when disease manifestations appear relatively stable. With regard to safety, all patients who enrolled in the extension study were successfully reintroduced to SA treatment after discontinuing treatment at the end of study LAL‐CL01.

The findings of this study are consistent with the phase 3, placebo‐controlled ARISE study, which showed that early improvements in transaminase levels and lipid profiles and reductions in liver fat content were maintained over long‐term treatment.^17^ In addition, in a study in a population of children and adults with a more broad presentation of LAL deficiency (LAL‐CL06), SA treatment for 96 weeks achieved sustained improvements in markers of liver and lipid dysfunction and improvements in liver and spleen volume, and was well tolerated.^18^

Limitations of this study include its open‐label design and its small sample size, which is consistent with a rare disease study and made determination of any dose‐related effects difficult and made statistical analyses not possible for all outcomes (*P* values were computed if n ≥6; considered descriptive, not confirmatory). Despite obvious improvement in the lipid profile caused by SA, more than half of patients (75%) were receiving concomitant lipid‐modifying medications during the study, which may have had confounding effects on study outcomes. However, during the washout period between LAL‐CL01 and LAL‐CL04, increases in LDL‐C levels and decreases in HDL‐C levels were observed,^15^ which seems to underline the positive impact of SA treatment, regardless of concomitant use of lipid‐modifying medications. Because there has been no trial of removing the lipid‐modifying medications, it is unknown whether these medications are needed to provide additional improvements in lipid parameters beyond those conferred by SA in patients with LAL deficiency. Another limitation is that noninvasive assessment of liver fibrosis is lacking in this study; however, surrogate biomarkers of liver fibrosis/cirrhosis (APRI and FIB4), although not validated in LAL‐D, indicated a decrease in fibrosis/cirrhosis from baseline to end of study. In addition, change in cardiovascular disease risk was not a prespecified outcome in this study. As such, cardiovascular assessments, including carotid duplex/carotid intima‐media thickness scans or coronary artery calcium scans, were not performed before or during the study.

In conclusion, in adults with LAL deficiency, the early and rapidly achieved beneficial effects of SA were maintained throughout 5 years of treatment, with substantial and sustained improvements in serum transaminases, lipid abnormalities, and liver volume and fat content. Treatment with SA for up to 5 years was generally well tolerated. These findings support the long‐term efficacy and safety of SA treatment in adults with LAL deficiency.

## CONFLICT OF INTEREST

VM received funding for participation as a study investigator and an honorarium for chairing an educational session from Alexion Pharmaceuticals, Inc JBA and RS have received honoraria for educational sessions and funding from Alexion Pharmaceuticals, Inc for participation as study investigators. JK has given lectures on this topic supported by Alexion Pharmaceuticals, Inc, and has received funding from Alexion Pharmaceuticals, Inc for participation as a study investigator. MB is a member of the LALD scientific registry and has received honoraria for participation in advisory boards and funding from Alexion Pharmaceuticals, Inc for participation as a study investigator. CBW received consultancy fees and funding from Alexion Pharmaceuticals, Inc for providing education on lysosomal diseases. SM and FA are employees of and may own stock/options in Alexion Pharmaceuticals, Inc

## LINK TO DATA REQUEST FORM

https://alexion.com/contact‐alexion/medical‐information

## Data Availability

Alexion will consider requests for disclosure of clinical study participant‐level data provided that participant privacy is assured through methods like data de‐identification, pseudonymization or anonymization (as required by applicable law), and if such disclosure was included in the study's informed consent form or similar documentation. Qualified academic investigators may request participant‐level clinical data and supporting documents (the statistical analysis plan and study protocol) for Alexion‐sponsored studies. Further details regarding data availability and instructions for requesting information are available in the Alexion Clinical Trials Disclosure and Transparency Policy at http://alexion.com/research‐development.
